# Private Nurses' Fees

**Published:** 1921-02-26

**Authors:** 


*To the Editor of* The Hospital.

### Private Nurses' Fees.

*To the Editor of* The Hospital.

Sir,—I am the superintendent of a large private nurses' co-operation in the Midlands. I am much interested in your article of January 29, page 413, of The Hospital. Having had many years of experience in this branch, I consider it is a much better policy to keep nurses busy at a minimum fee of three guineas weekly for ordinary medical and surgical nursing than as an alternative there should be a longer waiting period between cases, and a "falling off" of work in consequence of any further increase.

During the war, when private nurses were scarce, the fee was raised one guinea per week, and they were kept busily employed. Since the war large numbers have returned to their various Associations—V.A.D.'s have also returned, and are already being employed by private patients.

I am of the opinion that it will be detrimental to the interests of private nurses if any further increase is made.

The "general public" is a difficult factor to deal with—there are many illnesses which can be dealt with by intelligent and domesticated women who are not trained, and there may be a chance of the trained women becoming, to a certain extent, the unemployed. The present burden of taxation and living is a serious drawback to any further advancement, and all right-thinking nurses will be wise to be satisfied with the present reasonable charges.

I find that of late years patients' friends much more frequently employ two nurses, so that neither nurse shall be overtaxed, and this adds considerably to the expense of the illness. Cases are of much shorter duration, nurses are released at the first possible moment, owing to expense, if there is any further increase in fees the position may become more difficult.—Yours faithfully,

"Looking Forward."

